# Arenavirus Evasion of Host Anti-Viral Responses

**DOI:** 10.3390/v4102182

**Published:** 2012-10-17

**Authors:** Melissa Hayes, Maria Salvato

**Affiliations:** Institute of Human Virology, University of Maryland School of Medicine, Baltimore, MD 21201, USA; Email: melissawhayes@gmail.com

**Keywords:** arenavirus, innate immune response, immune evasion, TLR2

## Abstract

The innate response to infection by an Old World arenavirus is initiated and mediated by extracellular and intracellular receptors, and effector molecules. In response, the invading virus has evolved to inhibit these responses and create the best environment possible for replication and spread. Here, we will discuss both the host’s response to infection with data from human infection and lessons learned from animal models, as well as the multitude of ways the virus combats the resulting immune response. Finally, we will highlight recent work identifying TLR2 as an innate sensor for arenaviruses and how the TLR2-dependent response differs depending on the pathogenicity of the strain.

## 1. Introduction

Old World arenaviruses cause a wide spectrum of disease in humans, determined by the strain of virus and the immune status of the patient. Lassa virus (LASV) affects an estimated 2 million people each year, causing mild, flu-like illness to severe multisystem disease. Lymphocytic choriomeningitis virus (LCMV) generally causes a mild, self-limiting illness in the immunocompetent person with rare progression to severe or fatal encephalitis, but can cause a hemorrhagic fever-like disease in patients in which the virus was acquired through solid organ transplantation, and are thus immunocompromised.

Because of strict regulations, studies involving LASV can be quite cumbersome. Since the earliest symptoms of LASV infection are non-specific, patient data is unreliable and incomplete. In order to understand the immune responses that contribute to LASV pathogenesis, or lack thereof, the use of surrogate arenaviruses, both Old and New World, in cell culture and animal model experiments helps to build a better picture of how Old World arenaviruses can cause severe disease.

## 2. Immune Response to the Old World Arenaviruses

### 2.1. Type I Interferon Responses

Interferons are generally the key cytokine in establishing both innate and adaptive antiviral responses [1,2,3,4]. However, Lassa virus (LASV) is a poor inducer of IFN-I, is relatively resistant to IFN (α and γ), and IFN-γ sensitivity of LASV isolates from human patients has not been shown to correlate with disease progression [5]. It has been demonstrated experimentally that purified LASV RNA can stimulate IFN-β production in a 5’ triphosphate dependent manner via RIG-I, however, this result has not been confirmed during virus infection, either *in vitro* or *in vivo* [6]. Human macrophages infected with Mopeia virus (MOPV), a non-pathogenic relative of LASV, *in vitro* produced significant amounts of IFN-I [7]. Using LASV-virus like particles (VLPs) expressing GPC and Z proteins or Z alone, there was a decrease in VLP release when tetherin, an interferon stimulated gene, was expressed [8].

In the natural host, the mouse, the nucleoprotein (NP) of LCMV associates with MDA5 and RIG-I, cytosolic innate receptors belonging to the RIG-like Helicase (RLH) family, to induce IFN-I, in conventional dendritic cells (cDC) but not in plasmacytoid dendritic cells (pDC) [9]. *In vivo* analysis of these results in the serum of mice showed RIG-I and MDA5 is involved in sustained IFN-I responses. However, interferon-β promoter stimulator gene (IPS-1, also called MAVS or CARDIF), an adaptor protein for RIG-I and MDA5, was not necessary for the IFN-I response as IFN-β production in IPS-1^-/-^ mice was similar to WT in lymphocytic choriomeningitis virus (LCMV)-infected mice and only modest impairment of IFN-α was observed. Taken together, RLHs are unlikely to be the primary pathogen recognition receptor (PRR) responsible for human innate antiviral defenses to Old World arenaviruses, since IPS-1 is their required adaptor molecule [10]. 

However, the relevance of any IFN-I activity is still in question as *in vitro* treatment of LCMV and LASV with IFN-α only reduced viral replication by 1 or 2 logs (Vero cells or Huh-7 cells respectively), and yet maintained the specific infectivity compared to untreated virus [5]. Antagonism of IFN-I responses to LASV or LCMV via the nucleoprotein in rodents likely contributes more to establishing and maintaining a persistent infection [11]. 

### 2.2. Proinflammatory Responses

The induction of innate proinflammatory cytokines and chemokines may play an interesting role in the control of arenavirus infection as there appears to be a correlation between fatal LASV infection and an absence of proinflammatory cytokine/chemokine responses in patient serum, whereas these cytokines are detected in survivors [12]. The absence of the prototypic proinflammatory CXC chemokine, IL-8 (CXCL8), was most strongly correlated with fatal outcome. IL-8 enhances leukocyte adherence to endothelium cells, and directs extravasation and migration of leukocytes into virus infected tissues [13]. *In vitro*, it was demonstrated that MOPV, but not LASV, was able to induce expression of IL-8, as well as other cytokines, in monocyte-derived macrophages and endothelial HUVEC cells [14]. 

The divergent induction of these cytokines may occur as a result of differences in transcription factor activation. Using a New World arenavirus model for LASV pathogenesis, guinea pigs infected with a virulent strain of Pichinde virus (PICV) induced the suppressive NFκB homodimer, p50/p50, while the non-virulent PICV strain induced the activating heterodimer p65(RelA)/p50 [15]. In another study, a thioaptamer decoy of AP-1 proteins was able to induce cytokine expression during virulent PICV infection and reduced virus-mediated mortality *in vivo*, validating the importance of proinflammatory cytokines in protection against arenavirus infection [16].

**Figure 1:**
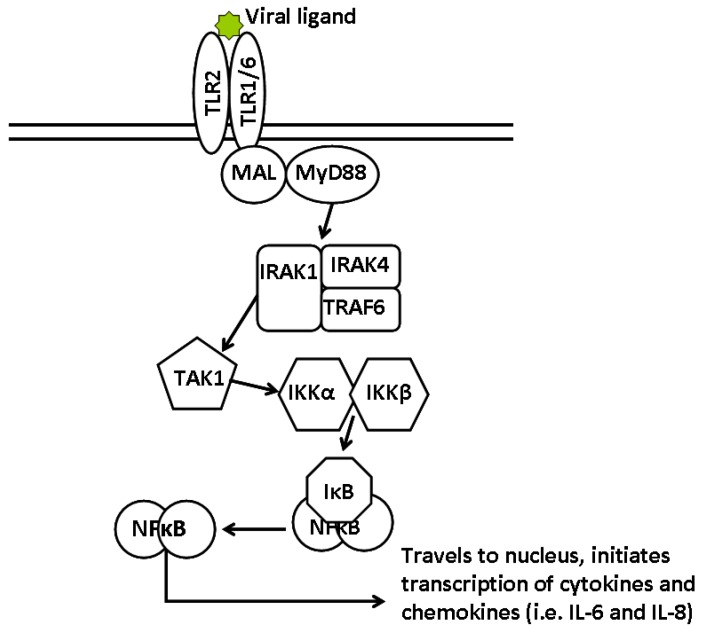
Signal transduction pathway from viral stimulation of TLR2 to induction of IL-8 through NFκB. Agonist binding to TLR2, along with a co-receptor, TLR1 or TLR6, either on the cell surface or within an endosome, leads to release of NFκB through degradation of the IκB inhibitor. Activation of the NFκB transcription factor allows for translocation to the nucleus and induction of proinflammatory cytokines, such as IL-8 and IL-6.

Toll-like receptor 2 (TLR2) has been shown to respond to LCMV infection. Generally, TLR2-ligand interactions generate proinflammatory cytokine responses via activation of NFκB (Figure 1). *In vitro* (IL-8), and *in vivo*, (IL-6 and MCP-1) cytokine responses to LCMV infection were found to be TLR2- and MyD88-dependent [17]. Unexpectedly, IFN-I production in mice was also found to require TLR2, but not MyD88. Recent studies have shown that some viral ligands are able to induce IFN-I production through TLR2 in the endosome, while bacterial ligands could not, demonstrating the plasticity and specificity of TLR2 functions [18]. Further, the proinflammatory responses observed during LCMV infection of CNS glial cells was determined to be TLR2-, MyD88-, and Mal-dependent [19]. TLR2, TLR6, and Mal were determined to be necessary for the proinflammatory cytokine response in THP-1 cells infected with the Armstrong strain of LCMV (LCMV-ARM) [20].

### 2.3. Adaptive Immune Response

For Old World arenavirus clearance, it is well accepted that a cytotoxic T-cell response is the primary mechanism [21]. Proliferative CD4^+^ T cell responses have been detected in seropositive people to epitopes mapped to the nucleoprotein and glycoprotein. The NP-specific responses have been documented in persons having survived LASV infection at least six years prior, demonstrating that long term memory is possible [22,23]. Notably, MyD88-deficient mice are unable to control viral replication or spread in a manner that does not involve IL-1R1, IL-18R, or IPS-1, indicating that this adaptor molecule plays a role in host defenses beyond the typical innate immune response [10,17,24,25]. MyD88 has been described as an important T-cell intrinsic factor to support expansion and survival of LCMV-specific CD8 T cells [26]. While WT CD8^+^ and CD4^+^ T cells expand and function in a MyD88^-/-^ host, MyD88^-/-^ CD8^+^ and CD4^+^ T cells have impaired functionality when transferred into a WT host, leading to a reduced cytotoxic T cell response to LCMV infection [10,22,26]. While T cells are able to be activated and expand, the sustained expansion and accumulation of LCMV-specific T cells is defective in the absence of MyD88 signaling. This appears to be an intrinsic requirement of T cells, as MyD88 deficient antigen presenting cells (APCs) are able to effectively activate WT T cells and adoptive transfer of MyD88 deficient T cells into a WT background are defective as described above [24,25,26]. Though fewer LCMV-specific T cells develop in the absence of MyD88, memory cells are generated from this smaller pool [26]. LCMV-specific memory T cells do not require the presence of MyD88 to maintain homeostasis or mount an effect response to secondary infection [25,27]. This requirement for T-cell intrinsic MyD88 signaling may be more important in chronic infection for prolonged expansion when T cells are exhausted. 

Antibody responses generally are not thought to assist in Old World arenavirus clearance, whereas this is the main mechanism of protection against New World arenaviruses. Patients recovering from LASV infection generally have low and delayed humoral responses. Early and brief IgG and IgM responses have been documented early in illness, but do not correlate with viral clearance as high viremia and high antibody titers can co-exist [21,28]. Neutralizing antibodies are undetected in patients during early convalescence and are only observed in low titers in a minority of surviving patients several months after clearance of infection [28].

## 3. Immune Evasion

Severe LASV infection is characterized by unchecked viremia, functional liver damage, and immunosuppression [29,30]. Viremia is strongly linked to disease manifestation and fatal outcomes. Viral evasion of immune mechanisms that would limit replication and spread would be beneficial to the virus. Inhibition of the antiviral state in infected cells as well as undiscovered infection by innate immune effector cells would allow efficient replication and dissemination. A lack of cytokine induction has been documented with pathogenic LASV and LCMV, strain WE [14,31,32]. In patients succumbing to LASV infection, the absence of IL-8 and IP-10, among a few other cytokines, was highly correlated to fatal outcome [12]. Exposure of PBMC from healthy donors to LASV demonstrated that IFN-related and apoptotic genes, as well as NFκB and coagulation pathways were the most highly affected in gene expression analysis [33]. However, treatment with IFN-I, but not IFN-γ, only limited arenavirus replication 10-fold in DC, and 10-100 fold in macrophages [34]. In accordance with the minimal effect of IFN-I on viral replication, it has been well documented that the nucleoprotein of all arenaviruses, with the exception of Tacaribe (TACV), functions as an IFN-I antagonist [35,36,37]. It is possible that the regulation of IFN-I by NP provides for chronic infection of the virus in its natural host as both virulent and non-virulent arenaviruses antagonized IFN-I in a dose-dependent manner [36].

Additionally, arenaviruses have a specific tropism for APCs, in which they are able to infect without activating these cells [7,38,39]. In human DC:T-cell co-cultures, LASV induced only weak memory phenotype markers, while MOPV strongly stimulated CD8^+^ and CD4^+^ T cells, activation markers, proliferative responses, and cytotoxic lymphocyte (CTL) activities [38]. We can speculate that TLR2-mediated production of cytokines in MOPV-infected cells [14] contributes to maturation of APC and thus strong adaptive immune responses. With 99% of the host cellular receptor, α-dystroglycan (DG), being expressed in the spleen, the virus is able to infect large numbers of APCs without initiating hallmark activation signals [34,39,40]. Infected DCs do not undergo migration or upregulation of costimulatory molecules thus allowing for efficient viral replication. These infected DCs may provide a reservoir of virus particles that can hide from immune surveillance. Infected macrophages lack phagocytic abilities and do not upregulate cytokines or chemokines that would recruit additional effector cells. 

Collectively, the absence of cytokine production and the delayed maturation of APC potentially contributes to defective adaptive immune responses. The reduced expression of co-stimulatory molecules, CD40, CD80 and CD86, as well as down regulation of MHC class I and II molecules likely contributes to the observed lack of activated CD8^+^ and CD4^+^ T cells during infection in non-human primates and hence, an absence of cytotoxic function in mice [40,41,42,43,44]. However, while APCs fail to mature during arenavirus activation, they are still able to respond to external stimuli. Cytokines and costimulatory markers were upregulated even in infected cells when treated with LPS or poly:IC. Such treatment even inhibited viral replication, indicating that the cellular anti-viral mechanisms were not irreversibly damaged during infection [34].

Whether the inhibited induction of cytokines is a direct or indirect outcome of virulent virus infection, the lack of innate stimulation likely positions the host to have a delayed adaptive immune response that would effectively clear the infection. Suppression of cytokine responses, whether IFN-I or proinflammatory, might be necessary, but insufficient contributions to manifestation of disease. Pathogenesis may rely on other factors, such as viral replication, though replication of pathogenic and nonpathogenic strains was equivalent *in vitro* [14].

While a robust cell mediated immune response is observed *ex vivo* from patients surviving LASV infection, lack of efficient maturation of APCs and initiation of the adaptive CTL response may contribute to fatal LASV infections. Early responses to LASV infection are likely key to establishing a successful immune response. Given that IFN-I is poorly induced by LASV and that, with the exception of TACV, all arenaviruses encode an IFN-I antagonist, the proinflammatory immune response is likely necessary for establishing the ideal environment to combat arenavirus infection and limit viral replication. TLR2 is necessary for the proinflammatory response to LCMV *in vitro* and *in vivo*, however, the induction of proinflammatory responses by arenaviruses of different pathogenic potential has not been thoroughly examined.

## 4. Arenaviruses and TLR2

With the identification of TLR2 as a mediator of proinflammatory responses to arenavirus infection, a closer investigation into the role of TLR2 in arenavirus pathogenesis is warranted [10,17,19]. The role of TLR2-dependent proinflammatory responses in pathogenesis, may be a general feature of all arenaviruses, not just Old World arenaviruses, underscoring its importance. A recent publication demonstrated that the vaccine strain of Junin virus (JUNV), Candid 1, elicited TLR2-dependent proinflammatory cytokines in murine macrophages [45]. Another group comparing a pathogenic strain of JUNV to closely related, but non-pathogenic Tacaribe virus (TCRV), demonstrated that TCRV, but not pathogenic JUNV, induced high levels of proinflammatory cytokines in mice [46]. Thus, the increasing body of evidence suggests that an early induction of cytokines enhances innate immune responses and promotes effective adaptive immune responses to prevent the development of hemorrhagic fever during arenavirus infection.

The mechanism by which TLR2-dependent cytokine responses are induced during arenavirus infection, and conversely, how those responses are inhibited are unclear. We have examined the contribution of cellular and viral factors to gain insight into these mechanisms. Importantly, it is not yet known which arenaviral gene or genome segment is essential for TLR2 signaling.

### 4.1. Inhibition of the Proinflammatory Response

**Figure 2:**
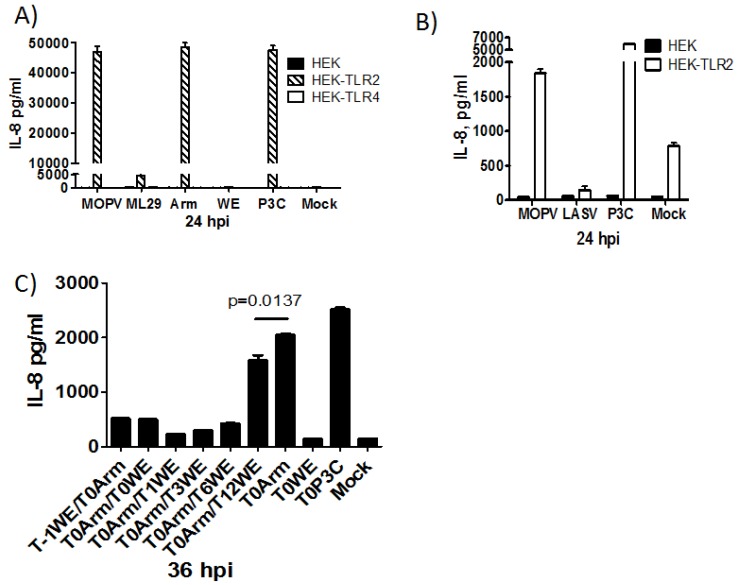
HEK293T cells or HEK293 cells stably expressing TLR2 or TLR4/MD2 were infected with MOPV, ML29, LCMV-ARM, and LCMV-WE (**A**), or with MOPV and LASV (**B**). Control cells were treated with media (mock infection) and with Pam_3_CSK4 (P3C) as a positive TLR2 control. At 24 hpi culture supernatants were removed and analyzed by IL-8 ELISA. (**C**) THP-1 cells were infected with LCMV-WE one hour prior to, simultaneously, or 1-12 hours post LCMV-ARM infection. Time points are based on LCMV-ARM infection at time zero (T_0_). T_-1_WE/T_0_ARM indicates WE infection one hour prior to ARM infection. Single infection controls, mock, and Pam_3_CSK4 controls were conducted at T_0_. At 13 hours post T_0_ infection, culture medium for all conditions was replaced with fresh media. Final culture supernatant samples were collected at 36 hours and analyzed for IL-8 production by ELISA.

Our laboratory recently investigated the TLR2-dependent responses of cells infected with arenaviruses of different degrees of pathogenicity (Figure 2) [20]. Non-pathogenic strains, such as MOPV and LCMV-ARM, induced robust levels of cytokines. However, pathogenic strains of LASV and LCMV-WE, the immunsuppressive LCMV-Clone13 (CL13), and to some extent the MOPV/LASV reassortant ML29, were unable to induce cytokine responses (Figure 2A-B). Infection with strains of differing pathogenic potential had similar effects on TLR2 expression at the cell surface, ruling out the possibility that the absence of cytokine responses were a result of differences in TLR2 expression. Strains of LCMV differentially activated NFκB, with LCMV-ARM eliciting significantly higher levels of NFκB reporter activation than LCMV-WE or LCMV CL13. Striking differences between LCMV-ARM and LCMV-CL13 are notable, given that there are only a few amino acid differences in their consensus sequences. 

TLR2-dependent proinflammatory responses are induced at the plasma membrane. However, we have demonstrated that UV or heat inactivated-LCMV is no longer able to elicit such a response. Additionally, multiplicity of infection, as well as replication, was strongly linked to the level of cytokine induction. Taken together, this data indicates that live replicating virus is necessary for efficient induction of proinflammatory immune responses. Further, because inactive virus was unable to induce TLR2-dependent responses, the possibility exists that arenaviruses stimulate secretion of a danger-associated molecules, capable of stimulating TLR2. Since live replicating virus is necessary for TLR2-dependent cytokine induction, it is probable that TLR2 stimulation occurs after membrane fusion and release of genomic material into the host cytoplasm. 

Co-infection experiments demonstrated the ability of pathogenic LCMV-WE to actively inhibit the cytokine response elicited by LCMV-ARM (Figure 2C) [20]. LCMV-WE was found to inhibit the cytokine responses most effectively up to 6 hours post infection, and partially up to 12 hours post LCMV-ARM infection. By this point in the virus life cycle, nucleoproteins, glycoproteins, and zinc proteins, as well as genomic and mRNAs are being synthesized. Any one of these components may be involved in suppressing the production of cytokines. Of the known viral TLR2 ligands, glycoproteins, core proteins, and nonstructural proteins are capable of stimulating TLR2 [47,48,49,50]. Just as live virus was necessary for TLR2-dependent cytokine induction, it was also necessary for inhibition, as UV-inactivated LCMV-WE failed to inhibit LCMV-ARM induced cytokine production. Similar to TLR2 stimulation, viral proteins or RNAs produced during replication may interrupt the NFκB activation pathway. 

In agreement with previous studies, LCMV-WE could not inhibit exogenous stimulation of TLR2-induced cytokine responses. The addition of LTA, a TLR2/6 ligand, in the presence of LCMV-ARM or LCMV-WE infection elicited cytokine production equivalent or greater than LTA treatment alone. This implies that the manner in which LCMV-WE inhibits the TLR2-dependent cytokine response is arenavirus-specific, or possibly specific to the type of ligand. It is possible that LCMV-WE may be capable of inhibiting TLR2-dependent cytokine production by other viruses. Further investigation with other viruses known to stimulate TLR2-dependent cytokine production in co-infection experiments with LCMV-WE will answer the specificity of LCMV-WE inhibition as ligand type plays an important role in TLR2 driven responses.

## 5. Future Directions

**Figure 3:**
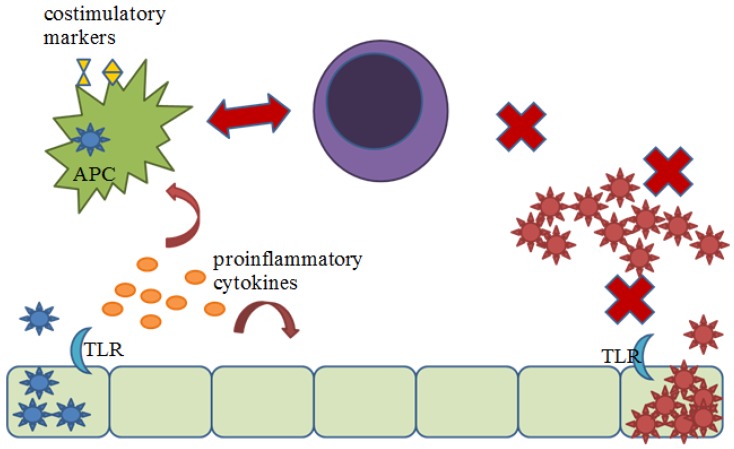
Proposed model for contribution of TLR2 signaling to protection against arenavirus infection. Non-pathogenic arenavirus (blue) infects host cells and begins to replicate, stimulating TLR2. TLR2-dependent signaling induced the production of cytokines creating an anti-viral environment, recruiting innate effector cells, and inducing the activation of APC. APC are then able to efficiently activate an adaptive cytotoxic T cell response, by presenting viral antigen and upregulating costimulatory molecules, which is able to clear the infection. Pathogenic arenaviruses (red) fail to stimulate TLR2, allowing for unrecognized infection and uncontrolled replication. APCs are not activated towards maturation and cannot effectively activate an adaptive cytotoxic response.

Two important questions remain: how does TLR2 signaling contribute to resistance/recovery from arenavirus infection and how could the virus inhibit TLR2 signaling? To determine the importance of TLR2 signaling in protection against arenavirus infection, the correct animal model must be used. Mouse models of arenavirus infection rely on IFN-I rather than inflammatory cytokines and cytotoxic T cell responses. These mouse models provide more information for chronic infections rather than severe acute infections, such as those caused by LASV in humans. The non-human primate model most closely resembles the disease progression seen in humans. However, methods to decipher the contribution of TLR2 are more difficult to pursue in such models. The use of function-blocking antibody treatment may provide some insight. Studies could then be directed toward the activation of APC, T cells, or viral load, in the absence or reduction of TLR2 signaling. These types of studies could help elucidate the contribution of TLR2 signaling in the model presented below (Figure 3), in which pathogenic arenavirus fails to create an antiviral environment during the early stages of infection broadly affecting the ability to elicit an efficient adaptive immune response.

**Figure 4:**
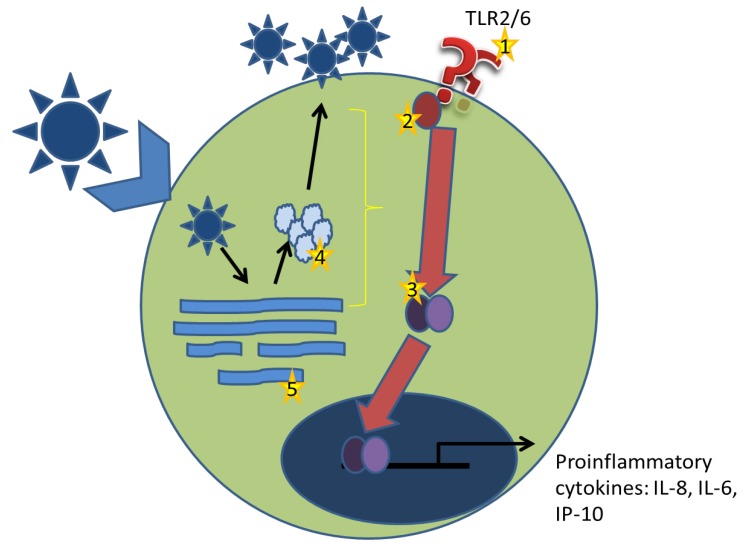
Working model for arenavirus induced TLR2 signaling. Arenaviruses bind the host cellular receptor and enter a host cell via receptor-mediated endocytosis. Through a unique pathway, arenaviruses enter the endosomal sorting complex and have released their genomic contents within 20 minutes. Replication of genomic and mRNAs as well as protein production begins. Virus particles are assembled at the plasma membrane and are released by budding. (1) In arenavirus-TLR2 stimulation, it is unlikely that the virus stimulated TLR2 from the plasma membrane as replication defective virus particles were unable to elicit a TLR2-dependent response. (2) Disruption of the signaling pathway could be achieved by preventing association of Mal with TLR2 TIR domain. During replication, the Glycoprotein complex inserts itself into the plasma membrane and has the potential to block association. (3) NFκB is differentially activated during infection with pathogenic and non-pathogenic arenaviruses. This indicates that the inhibition observed with LCMV-WE infection occurs upstream of NFκB activation. Given that viral replication was necessary for both induction of TLR2-dependent responses and inhibition, it is possible that a viral protein (4), or viral RNA (5) is exerting these effects. It is equally possible that viral replication induces a cellular stress response that stimulates TLR2.

Along with the global effects of TLR2 signaling in arenavirus infection, it is still necessary to determine how arenaviruses stimulate the TLR2-dependent response and how this is inhibited during pathogenic arenavirus infection (Figure 4). We have demonstrated the requirement for live replicating virus and the differential activation of NFκB. From these results, we hypothesize that stimulation of TLR2 occurs during the replication cycle of the virus, whether via a viral component such as a protein-derived ligand or through viral RNA recognition. It is also possible that during the replicative cycle of the virus, the stress induced on the host cell results in release of danger signals that could then stimulate TLR2 [51]. One possible method to determine the contribution of intracellular TLR2 signaling would be the use of intrabodies directed towards TLR2. Intrabodies are intracellular antibodies that are expressed and retained in the endoplasmic reticulum [52]. Recently, an intrabody directed towards TLR2 was reported that was derived from a monoclonal antibody antagonistic to human and murine TLR2. This intrabody was able to retain TLR2 from the plasma membrane effectively and prevent NFκB activation from the cell surface. Additionally, the use of cross-linking studies and molecular imaging would also be useful in determining how arenaviruses are stimulating TLR2. 

Ideally, one would like to singly express protein components of arenaviruses to determine if they stimulate TLR2. However, the requirement for live replicating virus confounds these efforts. The use of virus replicon particles (VRP) in which a replication defective virus particle encodes a gene under a separate promoter may help shed some light on this issue. VRPs could be used to express a particular gene of interest from either pathogenic or non-pathogenic arenaviruses. Additionally, using reverse genetics, proteins of pathogenic arenaviruses could be interchanged with the non-pathogenic protein, providing the replication necessary while singly expressing the pathogenic component.

By comparing the differences in TLR2-dependent responses during infection by arenaviruses of differing pathogenic potential for humans and non-human primates *in vitro*, we have provided a possible explanation for the development of severe disease in LASV infection. Further work in this area could entail looking for differences in TLR2 stimulation, TLR2 mutations, and TLR2-related SNPs to determine why some, but not all infected patients, succumb to LASV infection. Additionally, having identified the differences in TLR2-dependent cytokine responses between pathogenic and non-pathogenic virus infection, this work provides an avenue for development of therapeutics. With limited treatments available to use against LASV infection, use of TLR2 agonists may provide activation of the type of immune response necessary to clear virus more effectively and increase chances for survival. It is not clear whether activation of TLR2 signaling should be taken into account in vaccine design for arenaviruses. The ideal vaccine candidate for Old World arenaviruses would stimulate an efficient cytotoxic response and generate long lasting memory. Knowing the role of TLR2/Mal/MyD88 signaling in generating specific CD4^+^ and CD8^+^ T cell responses, and taking into consideration the inability of LASV to induce memory markers in T cells, it remains paradoxical that ML29, containing a small genomic segment from Lassa virus, has impaired induction of IL6 and IL8, yet is an excellent attenuated vaccine in non-human primates. Further reverse genetic and systems studies will be necessary to assess the role of the proinflammatory response in pathogenesis and protection.

## Supplementary Files

Supplementary File 1: PDF-Document (PDF, 567 KB)
