# Supplementary material for: Arenavirus Evasion of Host Anti-Viral Responses
Source: Viruses. 2012 Oct 17;4(10):2182–96. doi: 10.3390/v4102182 (PMC3497047; doi:10.3390/v4102182)
Supplement: Supplementary File 1: — PDF-Document (PDF, 567 KB) [file viruses-04-02182-s001.pdf]

Supplementary Data

1. Patient Data

The Retrovirus cohort represents patient samples diagnosed with either HIV or HTLV infection of which samples (plasma and serum) were stored from 1984 to 1995 at the Tygerberg hospital in Cape Town, South Africa. The patient, a South African coloured (mixed race) male born on 22 August 1931 was diagnosed with lymphocyte depleted Hodgkin's lymphoma on 02 March 1989 and diagnosed as HIV-1 positive on 09 March 1989. He travelled frequently to Lusaka, Zambia, where he possibly became infected with the virus.

Subsequently, serum and peripheral blood mononuclear cells (PBMCs) were obtained during November 1989 (harvested on 20 and 21 November 1989) and the virus was co-cultured with PBMCs and isolated. High molecular weight DNA was extracted from the HIV positive cultures through phenol-chlorophorm extraction and stored. HIV-1 positive cultures were confirmed by reverse transcriptase (RT) assay that ranged from 12,495 to 35,073 counts per minute per milliliter (cpm/mL). The *env* gene was amplified by PCR, sequenced and identified as subtype C [1].

2. Addition of 5'-U3 with CMV-IE-Promoter

(Primers in Supplementary Table S1)

Supplementary Table S1. Primers used during the study.

**Primers**	**Sequence (5'-3')**	**Tm (°C)**	**Position relative to HXB2**
HIV_NgoMIV_F	GAATGCCGGCTGGATGGGCTAGTTTACTCCAAGAGAAGGCAAG	71	−129
CMVstart_NgoMIV	GAATGCCGGCTAGTTATTAATAGTAATCAATTACGGGTC	63	−129
CMF_overlap_F	CAGAGCTGGTTTAGTAACCGGGTCTCTCTAGGTAGACCAGATCTGAGCCCGGGAGCTC	77	R-start
CMV_overlap_R	GTGCTCCCGGGCTCAGATCTGGTCTACCTAGAGAGACCCGGTTACTAAACCAGCTCTG	77	R-start
SpeI-R	CTATTTGTTCCTGAAGGGTACTAGTGTTCCTGCTATG	64	1507
SpeI-F	CATAGCAGGAACTACTAGTACCCTTCAGGAACAAATAG	64	1507
PacI-R	CTCTAATTCTTTTAATTAACCAGTCTATTTTTC	54	6198
PacI-F	GAAAAATAGACTGGTTAATTAAAAGAATTAGAG	54	6198
BspEI-R	GTCTTTGTAATACTCCGGATGTAGCTCGCG	63	9393
BspEI-F	CGCGAGCTACATCCGGAGTATTACAAAGAC	63	9393
NotI-R	GAGCGGCCGCACTACCAAAAAGGGTCTGAGGGATCTCTAGTTAC	72	9700

All PCR amplifications during this study were done with the stable proofreading Herculase II polymerase (Stratagene). Briefly, the HIV-1 subtype C promoter was replaced and cloned into the pMJ4 vector by overlapping PCR, using restriction sites (Supplementary Figure S1). The 600 bp CMV promoter region was amplified from pEGFP-C1 with primers CMVstart_NgoMIV (5'-GAATGCCGGCTAGTTATTAATAGTAATCAATTACGGGTC-3'), containing a *NgoM*IV restriction site, underlined in the sequence and CMV_overlap_R (5'-GTGCTCCCGGGCTCAGATCTGGTCTACCTAGAGAGACCCGGTTACTAAACCAGCTCTG-3'),

containing the last 30 bp of the CMV promoter and the first 30 bp from HIV-1 subtype C transcription start (R-region). The 1.0 kb HIV-1 subtype C region from R-start to the SpeI site in gag was amplified from pMJ4 with primers CMF_overlap_F (5'-CAGAGCTGGTTTAGTAACCGGGTCTCTCTAGGTAGACCAGATCTGAGCCCGGGAGCTC-3') and HIVC_SpeI_R (5'-CTATTTGTTCCTGAAGGGTACTAGTGTTCCTGCTATG-3'), the *Spe*I site is underlined. Primers CMVstart_NgoMIV and HIVC_SpeI_R were then used to PCR amplify the 1.6 kb fragment and cloned directly into pMJ4. The presence of the CMV promoter was confirmed by DNA sequencing. The resulting plasmid was abbreviated as pcMJ4.

3. Cloning of pZAC

We first replaced the *env* of MJ4 with that of our primary isolate, R3714/ZAC using standard cloning techniques. The 3.2 kb PCR product was amplified from the HMW DNA of ZAC with primers containing the restriction enzyme recognition sites for *Pac*I and *BspE*I. This corresponds to position 6198 and 9393 relative to the reference HXB2 genome. Clones were screened by restriction enzyme digestion and sequenced to confirm the presence of the correct insert. The 5' fragment of ZAC was amplified in two further parts encompassing the *gagpol* and LTR-*gag* region. The *gagpol* region was replaced using restriction sites *Spe*I (corresponding to position 1507 of HXB2) and *Pac*I (corresponding to position 6198 of HXB2), while the CMV-IE LTR-*gag* sequence from ZAC was added as for pcMJ4. The new proviral clone was designated pcZAC. The 3'-U5 was replaced using *BspE*I and the vector located *Not*I restriction site and the 5'-U3 CMV-IE was replaced with the ZAC derived 5'-U3 sequence. The final clone (without the CMV-IE promoter) was named pZAC.

4. Vpu Expression Plasmids

The primers used to amplify the *vpu* genes from NL4-3, MJ4 and ZAC is listed in Supplementary Table S2. The genes were cloned into the pCDNA3.1 (Invitrogen) with restriction enzymes *BamH*I and *Xho*I, restriction sites are underlined in Supplementary Table S2. Hybrid clones of ZAC and MJ4 *vpu* were made through overlapping PCR using combination of the *BamH*I, *Xho*I primer pairs and primers PacI_F_MJ4vpu, PacI_R_MJ4vpu, PacI_F_ZACvpu and PacI_R_ZACvpu.

Supplementary Table S2. Vpu primers.

**Primers**	**Sequence (5'-3')**	**Tm (°C)**
BamH1_NL4-3_vpu_F	CCGAGCTCGGATCCAGTACCCTTCACCATGCAACCTATAATAGTAGCAATAG	72
Xho1_NL4-3_vpu_R	GCCCTCTAGACTCGAGCTACAGATCATCAATATCCCAAGGAGCATG	71
BamH1_MJ4_F	CCGAGCTCGGATCCAGTACCCTTCACCATGATAGATTTACTAGCAAGAGTAG	72
XhoI_MJ4_R	GCCCTCTAGACTCGAGCTACAAATTATCCAAAAGCCTAAG	66
BamHI_ZAC_F	CCGAGCTCGGATCCAGTACCCTTCACCATGATTGATTTACTAGCAGGAGTAG	73
XhoI_ZAC_R	GCCCTCTAGACTCGAGTTACAAATCATAAGCATCCAAAAG	66
PacI_F_MJ4vpu	GAAAGATAGACTGGTTAATTAAAAGAATTAGGGAAAGAGC	62
PacI_R_MJ4vpu	GCTCTTTCCCTAATTCTTTTAATTAACCAGTCTATCTTTC	62
PacI_F_ZACvpu	GAAAAATAGACTGGTTAATTAAAAGAATTAGAGAAAGGGC	60
PacI_R_ZACvpu	GCCCTTTCTCTAATTCTTTTAATTAACCAGTCTATTTTTC	60

Supplementary Figure S1. Amino Acid alignment of Env gp120 of the HIV-1 subtype C infectious clones. The variable regions (V1-V5) are marked as well as the CD4 binding domain. pZAC has a shortened V1 loop and a slightly enlarged V4 loop, compared to that of pMJ4 and pHIV1084i.

```
                       Signal peptide
NL4-3       MRVKEKYQHL WRWGWKWGTM LLGILMICSA TEKLWVTVYY GVPVWKEATT TLFCASDAKA
ZAC         ---MGITRNC QQ-.-I--IL GFWM----NV MGN------- --------KA P---------
MJ4         ----GIPRNW QQ-.-I--SL GFW..I---V MGN------- -----R--K- ----------
HIV1084i    ---RGIQRNY PQ-.-I--IL GFL..--YNG MGS------- --------K- ----------
IN.D24      ---GGILRNC QH-.-I--IL GFWMF---NV VGN------- -----R--K- ------E---
Indie_C1    ---RGTLRNY QQ-.-I--VL GFWM----NG GGN------- --------K- --L-------   60

NL4-3       YDTEVHNVWA THACVPTDPN PQEVVLVNVT ENFNMWKNDM VEQMHEDIIS LWDQSLKPCV
ZAC         -ER------- ---------- ---I--E--- -K-------- -K-------- ---E------
MJ4         -EA------- ---------- ---IE-K--- ------E--- -D-------- ----------
HIV1084i    -ER----I-- ---------- ---L--E--- ---------- -D-------- ----------
IN.D24      -EK------- ---------- ---LD----- ---------- -D-----V-- ----------
Indie_C1    -ER------- ---------- ---I--G--- ---------- -D-----V-- ----------  120

                                V1                          V2
NL4-3       KLTPLCVSLK CTDLKNDTN. ...TNSSSGR MIMEKGEIKN CSFNISTSIR DKVQKEYAFF
ZAC         -------T-N --NYI-.... .......... ..DTT--T-D ----MT-EL- --RK--H-L-
MJ4         -------T-N -KNVTSK... ....DINITS NAEM-A-M-- -----T-EL- --KKQ---L-
HIV1084i    -------T-N ---V-S.... .......... ANSTSEDMR- ----VT-ERK -RKKL-Q-L-
IN.D24      -------T-E -NHVNITY-A TIHNATDQAS FNKTREQMR- ----VT-EL- --KKS---L-
Indie_C1    -------T-E -RNVSR.... ...NV--YNT YNGSVE---- ----ATPEV- -RK-RM--L-  180

NL4-3       YKLDIVPID. ........NT S...YRLISC NTSVITQACP KVSFEPIPIH YCAPAGFAIL
ZAC         --P----LNE N..FNSSA-Y -..E----N- ---A-R---- ----D----- ------Y---
MJ4         -------LTN ...DNASE-A -..E----N- D--T---S-- --T-D----- ------YV--
HIV1084i    -R-----LK. ...NSSSS-F -..E----N- ---TVS---- --N-D----- ------Y---
IN.D24      --I----LKE EKKNNSSE-N -SGH----N- ---A------ --T-D----- --T-------
Indie_C1    -G-----LN. ..KKNSSE-S -..E----N- ---A------ --T-D----- ------Y---  240

NL4-3       KCNNKTFNGT GPCTNVSTVQ CTHGIRPVVS TQLLLNGSLA EEDVVIRSAN FTDNAKTIIV
ZAC         ---------- ---N------ -----K---T ---------- --EII---E- I-N-V-----
MJ4         ---------- ---N------ -----K---- ---------- -KEII---K- I---V-----
HIV1084i    ---------S ---N------ -----K---- ---------- ---II---E- L-N-V-----
IN.D24      --KD-K---- ---S------ -----K---- ---------- --EII---Q- L-N-------
Indie_C1    ---------- ---N------ -----K---- ---------- -GEII---E- L-N-V-----  300

                                     V3
NL4-3       QLNTSVEINC TRPNNNTRKS IRIQRGPGRA FVTIG.KIGN MRQAHCNISR AKWNATLKQI
ZAC         H--E----V- ---G------ V--..---QT -FAT-EI--K I-E------E DQ--K--HRV
MJ4         H--E----E- ---G----R- V--..---Q- -YAT-DI--D I-A------E S---KI-YRV
HIV1084i    H-KDY---V- ---------- M--..---Q- -YAT-EI--- I-E------G S---N--QRV
IN.D24      H--E----I- ---------- ---..---QT -YAT-DI--- I--------G .---E--YNV
Indie_C1    H--Q----V- ---------- ---..---QT -YAT-DI--D I--------- D---E-_QRV  360

                                                        V4
NL4-3       ASKLREQFGN NKTIIFKQSS GGDPEIVTHS FNCGGEFFYC NSTQLFNSTW FNSTWSTEGS
ZAC         SE--E-H-P- .---K-GPPT ---L--T--- ---R------ -TSS---G-Y MRP.....N-
MJ4         SE--K-H-P- .---Q-D-PI ---L--T--- ---R------ -TSK---G-Y N........-
HIV1084i    KK--G-H-P- -T--D--P-- ---L--T--- ---R------ -TSK---G-S E.........
IN.D24      SR--A-H-P- .---N-TSP- ---L--T--- ---------- -TSV-----Y NHT-KQF.S-
Indie_C1    GK--A-H-H- .---K-AS-- ---L--T--- ---R------ -TSG---G-Y MPTYMPN.-T   420

                        CD4 domain
NL4-3       NNTEGSDTIT LPCRIKQFIN MWQEVGKAMY APPISGQIRC SSNITGLLLT RDGGNNNN..
ZAC         TGNTSNS--- -H-K------ ---G--Q--- ----A-N-T- K------I-- ----QT--..
MJ4         TGDTSNS--- -S-----I-- ---G--R--- -S--A-N-T- K--------- -----ETS..
HIV1084i    ....SNS--- ---K---I-- ---G--R--- ----A-N-T- K--------- -----G-G..
IN.D24      PYNDTNS--- IH-K---I-- ------R-I- ----A-N-T- K--------V -----TES..
Indie_C1    ESN.SNS--- I------I-- ------R--- ----A-N-T- T--------V H---IKE-DT   480

              V5
NL4-3       ..GSEIFRPG GGDMRDNWRS ELYKYKVVKI EPLGVAPTKA KRRVVQREKR
ZAC         --TN-T---A ---------- --------EV K---L---T- -----E----
MJ4         ---I-----A ---------- --------E- K---L----S -----E----
HIV1084i    --.T------ ---------- ---------- ----I----- -----E-G--
IN.D24      -NNT------ ---------- --------EV K---I---A- -----E----
Indie_C1    ENKT------ ---------- --------E- K-------A- -----E----  530
```

References and Notes

1. Engelbrecht, S.; Laten, J.D.; Smith, T.L.; van Rensburg, E.J. Identification of env subtypes in fourteen HIV type 1 isolates from south Africa. *AIDS Res. Hum. Retroviruses* **1995**, *11*, 1269–1271.
